# Illuminating the Neural Landscape of Pilot Mental States: A Convolutional Neural Network Approach with Shapley Additive Explanations Interpretability

**DOI:** 10.3390/s23229052

**Published:** 2023-11-08

**Authors:** Ibrahim Alreshidi, Desmond Bisandu, Irene Moulitsas

**Affiliations:** 1 Centre for Computational Engineering Sciences, Cranfield University, Cranfield MK43 0AL, UK; desmond.bisandu@cranfield.ac.uk; 2 Machine Learning and Data Analytics Laboratory, Digital Aviation Research and Technology Centre (DARTeC), Cranfield University, Cranfield MK43 0AL, UK; 3 College of Computer Science and Engineering, University of Ha’il, Ha’il 81451, Saudi Arabia

**Keywords:** aviation safety, convolutional neural network, deep learning, EEG, electroencephalogram, interpretability/explainability, machine learning, mental states classification, pilot deficiencies, SHapley Additive exPlanations

## Abstract

Predicting pilots’ mental states is a critical challenge in aviation safety and performance, with electroencephalogram data offering a promising avenue for detection. However, the interpretability of machine learning and deep learning models, which are often used for such tasks, remains a significant issue. This study aims to address these challenges by developing an interpretable model to detect four mental states—channelised attention, diverted attention, startle/surprise, and normal state—in pilots using EEG data. The methodology involves training a convolutional neural network on power spectral density features of EEG data from 17 pilots. The model’s interpretability is enhanced via the use of SHapley Additive exPlanations values, which identify the top 10 most influential features for each mental state. The results demonstrate high performance in all metrics, with an average accuracy of 96%, a precision of 96%, a recall of 94%, and an F1 score of 95%. An examination of the effects of mental states on EEG frequency bands further elucidates the neural mechanisms underlying these states. The innovative nature of this study lies in its combination of high-performance model development, improved interpretability, and in-depth analysis of the neural correlates of mental states. This approach not only addresses the critical need for effective and interpretable mental state detection in aviation but also contributes to our understanding of the neural underpinnings of these states. This study thus represents a significant advancement in the field of EEG-based mental state detection.

## 1. Introduction

The human brain, an intricate network of billions of neurons, is a dynamic system that constantly generates electrical activity. This electrical activity, which reflects the complex interplay of neural processes, can be measured and analysed using electroencephalography (EEG). Since the advent of EEG in the early 20th century, these signals have been extensively studied for their potential to provide insights into various cognitive states, mental conditions, and neurological disorders [1,2,3]. In recent years, the use of EEG in cognitive neuroscience has surged, driven by advancements in signal processing techniques and the development of portable and wearable EEG devices [4]. The non-invasive nature of EEG, its relatively low cost, high temporal resolution, and the possibility of real-time monitoring make it a particularly attractive tool for studying brain dynamics in various contexts [5,6]. One such context is the field of aviation, where understanding and monitoring the mental states of pilots is of paramount importance. The mental state of a pilot can significantly influence his decision-making ability, reaction times, and overall performance, particularly in high-stakes or stressful situations [7]. Therefore, the ability to accurately detect and classify different mental states based on EEG data could provide a valuable tool for enhancing safety and performance in aviation.

Different mental states are associated with different patterns of brain activity, which can be captured in the frequency domain of EEG signals as characteristics of power spectral density (PSD) [8]. These features represent the distribution of signal power over various frequency bands, such as delta (δ), theta (θ), alpha (α), beta (β), and gamma (γ), each of which is associated with different cognitive processes and mental states [9]. For instance, δ waves are typically associated with deep sleep or relaxation, θ waves with creativity and insight, α waves with relaxed alertness, β waves with active thinking and focus, and γ waves with higher mental activity and perception [9,10]. The analysis of these frequency bands can provide a window into the cognitive processes underlying different mental states, making them a valuable tool for mental state classification [8].

In recent years, the field of machine learning (ML), particularly convolutional neural networks (CNN), has made significant strides in the analysis of EEG data for mental state classification [11]. CNN models have demonstrated their efficacy in handling high-dimensional data, such as EEG signals, by automatically learning hierarchical representations from raw data. This ability to learn and extract salient features from raw data without the need for manual feature extraction is a significant advantage in EEG analysis, where the selection of appropriate features is often challenging [11,12,13].

However, while CNN models have shown promise in terms of performance, understanding the decision-making process of these models remains a challenge. To address this, we employ SHapley Additive exPlanations (SHAP) values, a powerful tool for interpreting machine learning models. SHAP values provide a measure of the contribution of each feature to the model’s prediction, thereby offering insights into the model’s decision-making process [14,15].

The novelty of this study lies in the comprehensive approach to mental state detection in pilots, extending the boundaries of EEG-based cognitive state detection in several significant ways:

1. It employs a one-dimensional CNN (1D-CNN) architecture optimised for EEG data, comprising five convolutional layers, thereby enhancing the model’s capability to capture both spatial and temporal features.
2. The model is trained on PSD features extracted from EEG signals, offering a robust and computationally efficient approach to mental state classification.
3. We introduce the use of SHAP values for result interpretability, enabling a nuanced understanding of feature importance for each mental state class.
4. The model is validated on the Attention-related Human Performance Limiting States (AHPLS) dataset, notable for its real-world applicability to high-stakes environments like aviation.

The EEG data was sourced from the AHPLS dataset, a rich and diverse dataset that has been used in several recent studies to understand and model human cognitive states [16,17,18,19,20,21]. The AHPLS dataset is unique in its inclusion of data from pilots under various mental states, namely channelised attention (CA), diverted attention (DA), startle/surprise (SS), and normal/no event (NE) states. This provides a robust and realistic dataset for training and testing our model. The use of such a dataset is a significant contribution to the field, as it allows for the exploration of EEG-based mental state detection in a high-stakes real-world environment, such as aviation [22].

This study aims to answer the following research questions:

1. How effectively can a 1D-CNN model trained on PSD features of EEG signals detect four mental states in pilots?
2. What are the key features of PSD that contribute to the successful detection of these mental states?
3. How does the performance of the model vary across different pilots and when trained on data from all pilots combined?

The answers to these questions will provide valuable insights into the potential of EEG-based mental state detection in aviation and other high-stakes environments and will guide future research in this area.

The paper is organised as follows: In Section 2, an overview of mental states and related research studies is presented. The proposed comprehensive approach in Section 3 describes the methods used in this study. Section 4 outlines the Experimental Setup, Section 5 presents the findings, and Section 6 presents a discussion of the results. Finally, the conclusion of the study is stated in Section 7.

## 2. Related Work

The application of ML techniques, particularly deep learning (DL), to the analysis of EEG data for the detection of mental states, has been a topic of significant interest in recent years. This section provides an overview of the key research in this area, highlighting the methodologies used, the results obtained, and the gaps that this study aims to address.

### 2.1. Previous Studies on EEG-Based Mental State Detection

Several studies have explored the use of EEG data for the detection of mental states. For instance, Başar et al. [9] found that γ, α, δ, β, and θ oscillations govern cognitive processes, suggesting that these frequency bands could be used to detect different mental states. Similarly, Klimesch [8] found that EEG α and θ oscillations reflect cognitive and memory performance, indicating their potential for mental state detection. Regarding the application of ML and DL methods, Giudice et al. [23] developed a 1D-CNN model to detect and discriminate between voluntary and involuntary blinking of the eye using EEG data, demonstrating the potential of DL techniques for such tasks. Mattioli et al. [24] proposed an approach based on a 10-layer 1D-CNN to classify four motor imagery (MI) and baseline states, which showed promising results in terms of performance. Similarly, Tabar et al. [25] model based on CNN and stacked autoencoders to classify EEG MI signals, demonstrating the effectiveness of DL models compared to ML models. Furthermore, Zorzos et al. [26] extracted time-frequency domain characteristics from the EEG signal to train on a shallow CNN model with three convolutional layers for the detection of mental fatigue, which showed promising results in terms of performance and interpretability.

In the context of aviation, Wu et al. [27,28] addressed the problem of obtaining the representation of the fatigue status feature and detecting the fatigue behaviour status of pilots via EEG signals. The authors decomposed the EEG signals of pilots into four frequency bands, namely δ, θ, α, and β, and used them to train a deep contractive autoencoder network, achieving a 91.67% performance accuracy. Furthermore, Cui et al. [29] developed a CNN model to detect driver drowsiness using EEG data, achieving an average accuracy of 78.35% in 11 participants. They also employed an interpretation tool to recognise the biological features of drowsiness states. Han et al. [30] proposed a multimodal approach to classify four mental states, namely distraction, workload, fatigue, and normal, using EEG, electrocardiogram (ECG), respiration (R), and electrodermal activity (EDA). They extracted PSD features from the EEG signals, which were used to train a CNN model, and trained a long-short temporal memory (LSTM) model on the other non-brain signals, achieving an average accuracy of 85.2%. Johnson et al. [31] used the average power of the frequency bands as features to detect task complexity levels in flight simulator experiments. In addition, Roza et al. [32,33] focused on detecting the pilot’s emotions, namely happy, sad, angry, scared, surprised, and disgust, using an artificial neural network (ANN) in simulated flights. Binias et al. [34] proposed an ML approach to discriminate between states of brain activity related to idle but focused anticipation of visual signals and reaction to them.

### 2.2. Gaps in the Existing Literature

While these studies have made significant contributions to the field, there are several gaps that this study aims to address. Firstly, many previous studies have focused on binary or multiclass classification problems, such as distinguishing between rest and task states or between different levels of cognitive load. However, less research has been conducted on the detection of specific mental states, such as CA, DA, SS, and NE states, particularly in the context of aviation.

Second, while DL models have shown promise in EEG analysis, understanding the decision-making process of these models remains a challenge. Many previous studies have focused on improving the performance of these models, but less attention has been paid to their interpretability. This is a significant gap, as understanding the features that these models consider important can provide valuable insights into the underlying cognitive processes associated with different mental states.

### 2.3. Previous Research on Detecting CA, DA, SS, and NE States

Several studies have explored the detection of specific mental states using EEG data. For instance, Harrivel et al. [17,18] recorded brain signals (i.e., EEG) and non-brain signals (i.e., ECG, R, and galvanic skin response (GSR)), capturing the attention-related pilot performance limiting states, including CA, DA, SS, and NE. The authors employed various ML techniques to perform binary and multiclass classification tasks in two different studies. Terwilliger et al. [16] attempted to discriminate between the normal state and an event state, where the CA, DA, and SS states are combined and named an event state. In previous research [20], we performed a multiclass classification task to attempt to predict the CA, DA, SS, and NE states. The main purpose of the study was to measure the impact of applying different preprocessing techniques on the performance of the ML model and to investigate the feasibility of concatenating EEG datasets recorded from different environment settings. It was found that the employment of pre-processing techniques has an impact on classification performance. Thus, an automated preprocessing approach was proposed to improve the signal-to-noise ratio and classification performance [19]. The proposed approach demonstrated the importance of preprocessing EEG data before training them in ML models. The attention-related pilot performance-limiting states are heavily class imbalanced. In a recent study [21], we evaluated the impact of employing various data resampling techniques on classification performance. It was discovered that the use of a combination of downsampling and oversampling techniques improves the performance of the ML models.

However, less research has been conducted on the detection of the specific mental states of CA, DA, SS, and NE states. These states are particularly relevant in the context of aviation, where pilots need to rapidly switch between different mental states in response to varying task demands. Understanding and detecting these states could have significant implications for safety and performance in aviation and other high-stakes environments.

### 2.4. Positioning of the Current Work

This study builds on the existing literature in several ways. Firstly, it focuses on the detection of specific mental states that are relevant in the context of aviation, addressing a gap in the existing literature. Second, it uses a 1D-CNN model, which has been shown to be effective in handling high-dimensional data such as EEG signals. This model is trained on PSD features of EEG data, which represent the distribution of signal power over various frequency bands and are associated with different cognitive processes and mental states.

Furthermore, this study addresses the need for model interpretability in EEG analysis by employing SHAP values. SHAP values provide a measure of the contribution of each feature to the model’s prediction, offering insights into the model’s decision-making process. This approach not only leverages the power of CNN models for EEG analysis but also addresses the critical need for model interpretability in this domain.

The EEG data used in this study are sourced from the AHPLS dataset, a rich and diverse dataset that has been used in several recent studies to understand and model human cognitive states. The AHPLS dataset is unique in its inclusion of data from pilots under various mental states, providing a robust and realistic dataset for training and testing our model. The use of such a dataset is a significant contribution to the field, as it allows for the exploration of EEG-based mental state detection in a real-world, high-stakes environment such as aviation.

In summary, this study extends the existing literature by focusing on the detection of specific mental states in pilots using a 1D-CNN model trained on PSD features of EEG data. It also addresses the need for model interpretability by employing SHAP values to identify the important features of each mental state. The use of the AHPLS dataset further enhances the relevance and applicability of this research in the field of aviation.

## 3. The Proposed Approach

**Figure 1:**
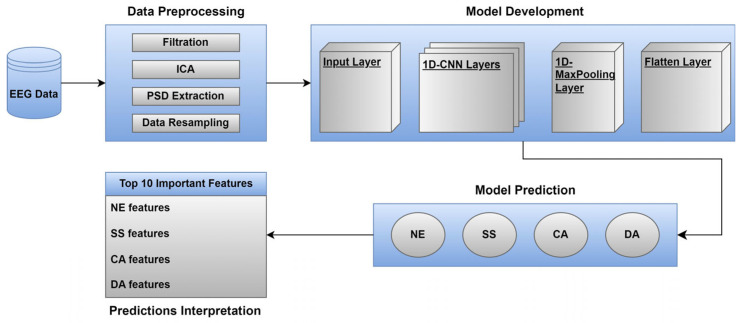
An overview of the proposed approach.

In this section, we describe the methods utilised to preprocess the EEG data, extract meaningful features from the EEG data, and handle the data imbalance issue. In addition, we explain the proposed 1D-CNN model and the interpretability method (i.e., SHAP) used to identify the most important features of each mental state. Figure 1 illustrates an overview of the proposed approach.

### 3.1. Data Preprocessing

EEG data was initially segmented into 1 s and filtered using a finite impulse response (FIR) filter with a frequency range of 1 to 40 Hz [35]. This step was instrumental in attenuating extraneous noise and enhancing the signal-to-noise ratio of the EEG data, thereby improving the quality of the data for subsequent analysis. Subsequent to the filtering process, the data were subjected to an artefact removal procedure to address ocular-related artefacts, a common occurrence in EEG data. This was achieved by utilising the Independent Component Analysis (ICA) algorithm, as delineated by Aapo Hyvärinen in his seminal work [36]. The ICA algorithm, renowned for its robustness in the separation of independent sources, was employed to isolate and subsequently remove components of the EEG data that were indicative of ocular movements.

Upon successful removal of artefacts, spectral analysis was performed on the sensor data. This was facilitated by the “multitaper” method, a technique that employs discrete prolate spheroidal sequences (DPSS) tapers [37]. This method was selected due to its ability to provide robust spectral estimates with minimised variance. The lower and upper-bound frequencies of interest were set to 1 and 40 Hz, respectively. This frequency range was strategically chosen to focus on the frequency bands that were pertinent to the study while concurrently excluding frequencies that could potentially introduce noise into the analysis. The rigorous preprocessing steps outlined above ensured the optimal preparation of the EEG data for the ensuing stages of the study.

Following the preprocessing of the EEG data, the dataset was partitioned into training and testing datasets, with proportions of 80% and 20%, respectively. Then, we split the partitioned training dataset into training and validation datasets, with proportions of 70% and 30%, respectively. This division was carried out to facilitate the model’s learning process and to ensure a robust evaluation of its performance. To address the issue of data imbalance, the SMOTEENN method was used. This hybrid resampling technique, which combines the synthetic minority oversampling technique (SMOTE) [38] and the Edited Nearest Neighbours (ENN), is highly effective in handling imbalanced data.

The SMOTEENN method first applies SMOTE to generate synthetic samples from the minority class, thereby balancing the class distribution. Mathematically, for each minority class sample x, it chooses one of its k nearest neighbours x’ and generates a new sample at a random point between x and x’, i.e., $x_{new}=x+\lambda .\;(x^{’}-x)$, where λ is a random number between 0 and 1. Subsequently, the ENN method is applied to remove any instances of the majority class that are surrounded by minority class instances and any instances of the minority class that are misclassified by its three nearest neighbours. This cleaning process ensures that the oversampling does not overgeneralise the minority class by creating noisy samples. The application of SMOTEENN in this study ensured a balanced representation of classes, thereby improving the model’s ability to generalise from the training data to unseen data.

### 3.2. The One-Dimensional Convolutional Neural Network (1D_CNN)

The 1D Convolutional Neural Network (1D-CNN) model is a variant of the traditional Convolutional Neural Network that is specifically designed for sequence data [39,40]. The model is composed of five 1D-CNN layers, followed by a MaxPooling1D layer and a flattened layer. The mathematical operation performed by a 1D-CNN layer can be described as follows.

Given an input sequence $x=[x_{1},x_{2},\;\ldots ,\;x_{n}]$, a filter $w=[w_{1},w_{2},\;\ldots ,\;w_{k}]$ of length k is applied to the sequence to produce a new sequence $y=[y_{1},y_{2},\;\ldots ,\;y_{n-k+1}]$, where each element $y_{i}$ is computed as

(1) $$y_{i}=b+\sum_{j=1}^{k}w_{j}\;\cdot \;x_{i+j-1}$$

Here, b is a bias term. This operation is applied for each filter in the layer, and the results are typically passed through a nonlinear activation function, such as the Rectified Linear Unit (ReLU) function.

After passing through the five 1D-CNN layers, a MaxPooling1D layer is applied. This layer reduces the dimensionality of its input by applying a max operation over sliding windows of a specified size. If the window size is p, then the output $z=[z_{1},z_{2},\;\ldots ,\;z_{n/p}]$ is computed as

(2) zi=maxj=i−1 p+1⁡yi

This operation helps to make the model more robust to shifts and distortions in the input data and reduces the computational complexity of subsequent layers. After passing through the five 1D-CNN and MaxPooling1D layers, the output is flattened into a one-dimensional vector. This flattened output can then be passed through one or more fully connected layers, which perform the final classification or regression task. The 1D-CNN model’s strength lies in its ability to effectively capture local dependencies in the input data, making it particularly well suited for tasks involving time series or sequence data. Its architecture allows it to learn both short- and long-term patterns in the data, which can be crucial for many prediction tasks.

### 3.3. SHapley Additive exPlanations (SHAP)

SHAP is a unified measure of feature importance that assigns each feature an importance value for a particular prediction. The concept of SHAP is based on Shapley values, a concept from cooperative game theory that assigns payouts to players depending on their contribution to the total payout [41]. SHAP values interpret the output of the ML and DL models using a game-theoretic approach, attributing the prediction of each instance to its features [15].

The SHAP value for a feature is the average marginal contribution of that feature across all possible combinations of features. Mathematically, the SHAP value φi for a feature i is given by

(3) $$\varphi i=\sum_{S\subseteq M\;\backslash \;\{i\}}\frac{(\left|S\right|\;!\left(\left|M\right|-\left|S\right|-1\right))!}{\left|M\right|!}\;\left[f\;\left(S\cup \{i\}\right)-f(S)\right]$$

where M is the set of all features, S is a subset of M without feature {i}, |S| is the number of features in S, |M| is the total number of features, and f is the prediction function. The term $(\left|S\right|!(|M|-|S|-1)!/|M|!)$ is the weight representing the number of times a subset S of size |S| appears in all possible subsets of M.

In this study, SHAP values are used to interpret the predictions of the CNN model trained on PSD features of EEG data. For each mental state prediction, SHAP values are computed for all features, providing a measure of the contribution of each feature to the prediction. This allows the identification of the top 10 most influential features for each mental state, offering insight into the neural correlates of these states. The use of SHAP values thus enhances the interpretability of the model, contributing to a deeper understanding of the neural mechanisms underlying the mental states of interest in aviation.

## 4. Experimental Setup

This section elaborates on the experimental setup employed in this study, encompassing the dataset, Python libraries, PC specifications, hyperparameter tuning, and evaluation metrics. Each component plays a crucial role in the overall research design and contributes to the validity and reliability of the results.

### 4.1. Dataset

In the present study, we employed a publicly released EEG dataset extracted from the AHPLS dataset. This dataset encompasses psychophysiological data derived from 20 EEG channels collected from 17 pilots operating within a flight simulation environment. The data were annotated with labels corresponding to different mental states, namely the CA, DA, SS, and NE states.

The EEG channels are denoted as follows: FP1, F7, F8, T4, T6, T5, T3, FP2, O1, P3, Pz, F3, Fz, F4, C4, P4, POz, C3, Cz, and O2. Each channel was sampled at a frequency of 256 Hz, ensuring a high-resolution temporal dataset.

A noteworthy characteristic of this dataset is its class imbalance. The NE class constitutes the majority of the dataset, accounting for 83% of the total instances. This is followed by the CA class, which comprises 14% of the dataset. The DA and SS classes are significantly underrepresented, making up 2% and 1% of the dataset, respectively. This class imbalance poses a challenge for conventional ML models, necessitating the use of specialised techniques to ensure robust and generalisable performance.

### 4.2. Python Libraries and PC Specifications

The computational experiments were conducted on a PC equipped with an Intel (R) Core (TM) i7-10700 CPU @ 2.90 GHz. The PC boasts a RAM capacity of 32.0 GB, ensuring efficient handling of large datasets and complex computations.

Python (version 3.10), renowned for its simplicity and powerful libraries, was used for all computational tasks. We utilised several Python libraries, each serving a distinct purpose. NumPy (version 1.21) and Pandas (version 1.3) were used for efficient data handling and manipulation, providing robust structures for dataset operations. MNE-Python, version 1.2, a library dedicated to processing electrophysiological signals, was used to handle the specific data types present in the AHPLS dataset. Scikit-Learn (version 1.0.2) was used for various ML tasks, including data preprocessing and model evaluation. TensorFlow, version 2.4, a powerful library for creating and training DL models, was used to construct and train our neural network models. Lastly, the SHAP library was used to interpret the predictions of the proposed framework.

### 4.3. Hyperparameter Tuning

In the process of model development, hyperparameter tuning was performed to optimise the performance of the 1D-CNN model. We utilised the Grid Search method to systematically explore a range of hyperparameters. The hyperparameters were fine-tuned based on the specific requirements of the task and the characteristics of the dataset. Table 1 summarises the hyperparameters that were fine-tuned for the 1D-CNN model:

The output layer of the model was a dense layer with four neurons and a softmax activation function, which is suitable for multiclass classification tasks.

In addition to the layer-specific parameters, several global parameters were also set for the training process. The learning rate was set to 1 × 10^−4^, which determines the step size at each iteration while moving towards a minimum of a loss function. The model was trained for 100 to 150 epochs, where an epoch is an iteration over the entire dataset. The batch size was set to 32, which is the number of samples processed before the model was updated.

The GlorotNormal initialiser was used with different seed numbers for initialising the kernel’s weights. This initialiser draws samples from a truncated normal distribution centred on 0, with stddev=sqrt(2/(fan_in+fan_out)), where fan_in is the number of input units in the weight tensor and fan_out is the number of output units. This initialiser is also known as the Xavier normal initialiser.

The hyperparameters were selected to optimise the model’s performance on the validation set, and the selected model was then evaluated on the test set to assess its generalisation capability.

### 4.4. Evaluation Metrics

The performance of the model was evaluated using a variety of metrics to provide a comprehensive assessment. Accuracy, the most intuitive performance metric, measures the proportion of correct predictions made by the model. Precision assesses the model’s ability to avoid false positives, measuring the proportion of true positive predictions among all positive predictions. Recall, on the other hand, evaluates the model’s ability to avoid false negatives, measuring the proportion of true positive predictions among all actual positives.

The F1 score provides a balance between precision and recall. It is particularly useful when the data have imbalanced classes, as it considers both false positives and false negatives in its calculation. A high F1 score indicates a robust model with a good balance between precision and recall.

In addition to these metrics, a confusion matrix was used to visualise the performance of the model. The confusion matrix provides a comprehensive view of how well the model performed across all classes, showing the true positives, true negatives, false positives, and false negatives. It is a powerful tool for understanding the model’s performance in greater detail, allowing for the identification of any classes that the model may be struggling to predict correctly.

Accuracy: This is the proportion of the total number of predictions that were correct. It is determined using the formula

(4) $$Accuracy=\frac{TP+TN}{TP+FP+TN+FN}$$

Precision: Also called the positive predictive value, this is the proportion of positive cases that were correctly identified. It is given by the formula

(5) $$Precision=\frac{TP}{TP+FP}$$

Recall: Also known as sensitivity, hit rate, or true positive rate (TPR), this is the proportion of actual positive cases that are correctly identified. The formula is as follows:

(6) $$Recall=\frac{TP}{TP+FN}$$

F1 score: This is the harmonic mean of precision and recall and tries to find the balance between precision and recall. It is given by the formula

(7) $$F1-score=2\times \frac{Precision\times Recall}{Precision+Recall}$$

## 5. Results

The results section of this study is organised into several subsections, each addressing a distinct aspect of the analysis. The first subsection investigates the impact of different mental states on the power distribution across EEG frequency bands. This analysis further elucidates the neural mechanisms underlying the mental states of interest and their manifestation in EEG data. The second subsection presents the performance metrics of the 1D-CNN model trained on the PSD features of EEG data from 17 pilots, along with the training accuracy, loss function curves, and the confusion matrix. These metrics include accuracy, precision, recall, and F1 score, providing a comprehensive evaluation of the model’s ability to detect four mental states: CA, DA, SS, and NE states. Lastly, the model interpretation subsection delves into the feature importance analysis, using SHAP values to identify the top 10 most important features for each mental state. This analysis provides insights into the EEG frequency bands and channels that are most influential in the model’s decision-making process, offering a deeper understanding of the neural correlates of the mental states under study.

Together, these subsections provide a comprehensive evaluation of the model’s performance, an exploration of the key features driving its predictions, and an examination of the neural underpinnings of the mental states it is designed to detect. The results presented in this section not only demonstrate the effectiveness of the proposed approach but also contribute to our understanding of the neural correlates of mental states in the context of aviation.

### 5.1. Examining the Effects of Mental States on EEG Frequency Bands

**Figure 2:**
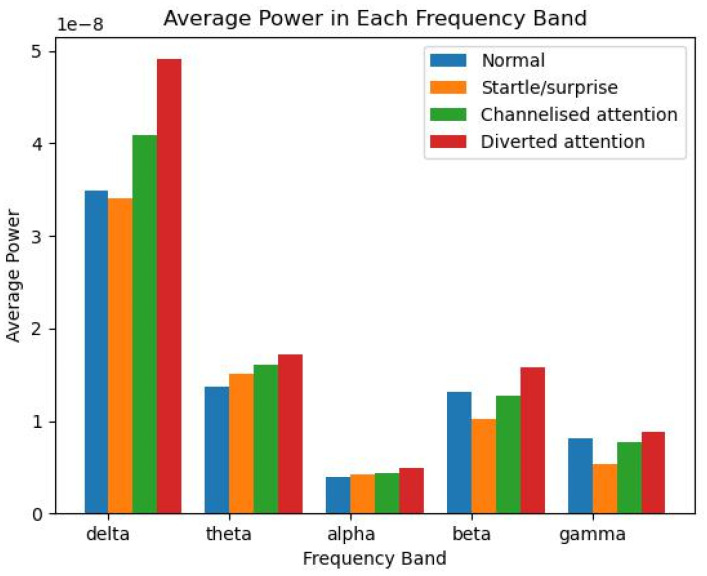
The average power in each frequency band across pilots.

**Figure 3:**
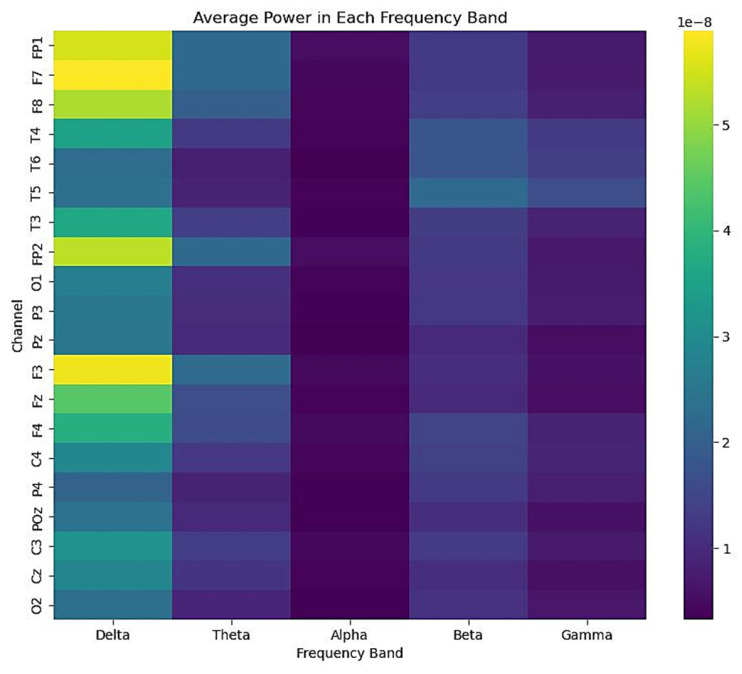
Heatmap for the average power in each frequency band for EEG channels.

The PSD of the EEG signals was analysed across different mental states and frequency bands. The average power in each frequency band (delta δ, theta θ, alpha α, beta β, and gamma γ) was calculated for each mental state (NE, SS, CA, and DA) and visualised using a bar plot shown in Figure 2 and a heatmap as depicted in Figure 3.

In Figure 2, the bar plot shows the average power in each frequency band for each mental state. The height of each bar represents the average power in that band for that state. It can be observed that there are distinct differences in the power across different frequency bands for each mental state. This suggests that the power in different frequency bands may be a useful feature for distinguishing between different mental states. However, there is also considerable variability within each band and state. This suggests that there may be individual differences or other factors that are not captured by the average power alone.

In Figure 3, the heatmap shows the average power in each frequency band for each EEG channel. The colour of each cell represents the average power in that band for that channel. It can be seen that there are distinct patterns of power across different channels and frequency bands. This suggests that the spatial distribution of power in different frequency bands may also be a useful feature for distinguishing between different mental states. However, it is also apparent that there is considerable variability across different channels, suggesting that the power in different frequency bands may be influenced by the location of the electrodes and the underlying brain regions.

Together, these results suggest that the power in different frequency bands and the spatial distribution of the power across different channels may be useful features to distinguish between different mental states. However, further analysis is needed to determine the statistical significance of these differences and investigate the potential influence of other factors, such as individual differences and electrode placement. Future research could also investigate the temporal dynamics of power in different frequency bands, as the current analysis only considers the average power over the entire recording period.

### 5.2. Classification Results

The study utilised the proposed model to identify four distinct mental states of the pilots: CA, DA, SS, and NE states. The model was trained on PSD features derived from five frequency bands across 20 EEG channels. This approach allowed for a comprehensive representation of the EEG data, capturing the complex interplay of different frequency bands across multiple channels.

The model was trained individually on each of the 17 pilots and then on the combined data of all pilots. The performance of the model was evaluated using four key metrics: Accuracy, precision, recall, and F1 score. These metrics provide a holistic view of the model’s performance, capturing its ability to make correct predictions (accuracy), its ability to correctly identify positive instances (precision), its ability to identify all positive instances (recall), and the balance between its precision and recall (F1 score).

As presented in Table 2, the results showed a high degree of consistency across all metrics for each pilot. The accuracy, precision, recall, and F1 scores all fell within a relatively narrow range of 94% to 99%. The highest accuracy and precision of 99% were achieved by Pilot 2. The lowest scores across all metrics were observed for Pilot 12, with an accuracy of 94%, precision of 91%, and an F1 score of 90%.

When the model was trained on the combined PSD features of all pilots, it achieved an accuracy, precision of 96%, recall of 94%, and F1 score of 95%. This suggests that the model was able to generalise well from individual pilots to a larger population.

**Figure 4:**
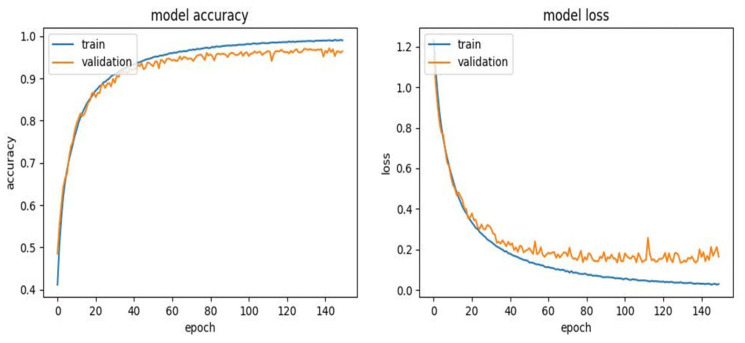
Training accuracy and loss curves of the proposed model.

In addition to the proposed model performance metrics, the training process was also evaluated by examining the accuracy and loss curves for the training and validation datasets, as depicted in Figure 4. For the training dataset, the accuracy curve demonstrated a consistent upward trend, indicating a steady improvement in the model’s ability to correctly predict the mental states as the training progressed. This consistent improvement suggests that the model was effectively learning the patterns in the training data and adapting its parameters accordingly. Simultaneously, the loss curve for the training dataset showed a consistent downward trend, indicating that the model was successfully reducing the error in its predictions over time. This is a positive sign of the model’s learning capability as it shows that the model was able to progressively minimise the discrepancy between its predictions and the actual values.

In contrast, the accuracy and loss curves for the validation dataset showed slight irregularities. Despite these irregularities, the overall trend of the validation accuracy curve was positive, and the validation loss curve generally showed a decreasing trend. This indicates that, despite the fluctuations, the model was able to apply what it learnt from the training data to unseen data, demonstrating a good level of generalisation.

**Figure 5:**
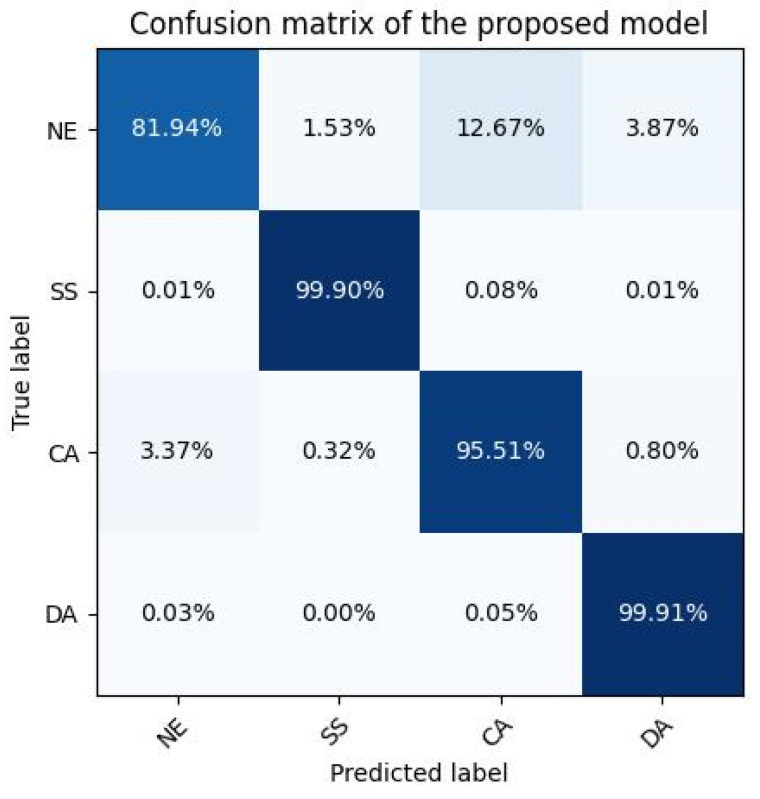
Confusion matrix of the proposed approach.

The performance of the proposed model was evaluated in a more detailed manner using a confusion matrix. This matrix provides a comprehensive view of the model’s ability to correctly classify each of the four mental states: NE, SS, CA, and DA. The matrix is structured such that each row represents the instances in an actual class while each column represents the instances in a predicted class. The confusion matrix is depicted below in Figure 5.

The diagonal elements of the confusion matrix, which represent the percentage of correct predictions for each mental state, show that the model achieved high accuracy rates for each of the four mental states, with the lowest being 81.94% for the NE and the highest being 99.91% for the DA state.

In a rigorous comparative analysis, the performance of the proposed framework was benchmarked against various established neural network models outlined in existing literature. The models chosen for this comparative study encompassed Deep Contractive Autoencoder Networks (DCAEN), ANN, CNN, and LSTM. The empirical evaluation revealed that our model exhibited superior classification accuracy. Specifically, the proposed model surpassed the DCAEN model by 21%, the CNN model by 12%, the LSTM model by a substantial margin of 16%, and the ANN model by 16%. This quantitative advantage underscores the efficacy of the proposed framework in learning and generalising from the data, thereby outclassing the comparative models in terms of performance. The comparative performance metrics are succinctly tabulated below in Table 3.

### 5.3. Model Interpretation Using SHAP

The study employed SHAP values to identify the top 10 most important features for each mental state class: NE, SS, CA, and DA. The SHAP values provide a measure of the contribution of each feature to the model’s prediction for each class, allowing for an understanding of which features are most influential in determining the mental state.

**Figure 6:**
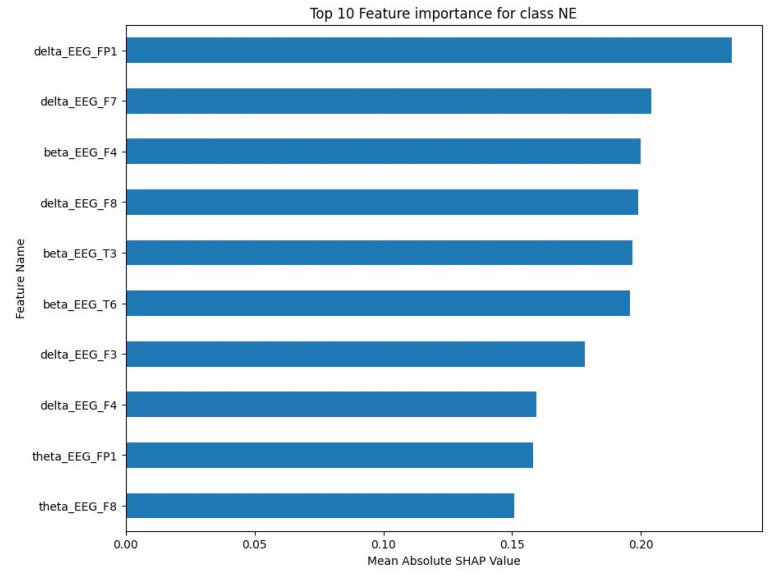
Top 10 important features for NE class.

**Figure 7:**
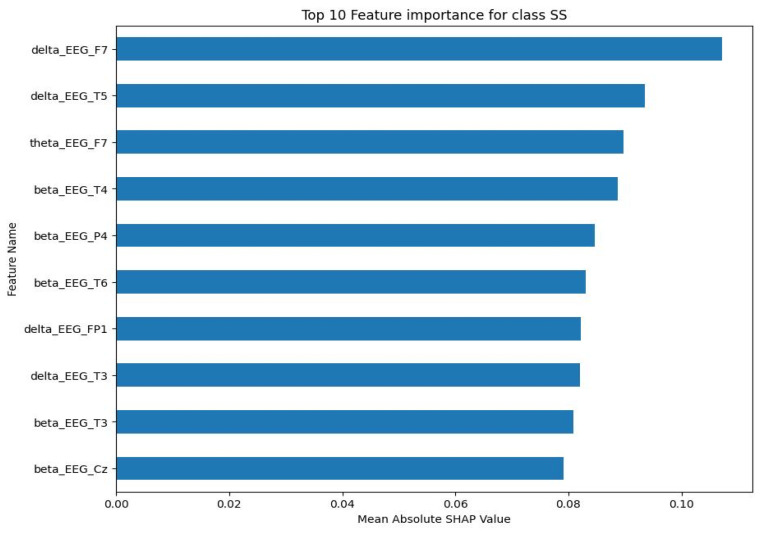
Top 10 important features for SS class.

**Figure 8:**
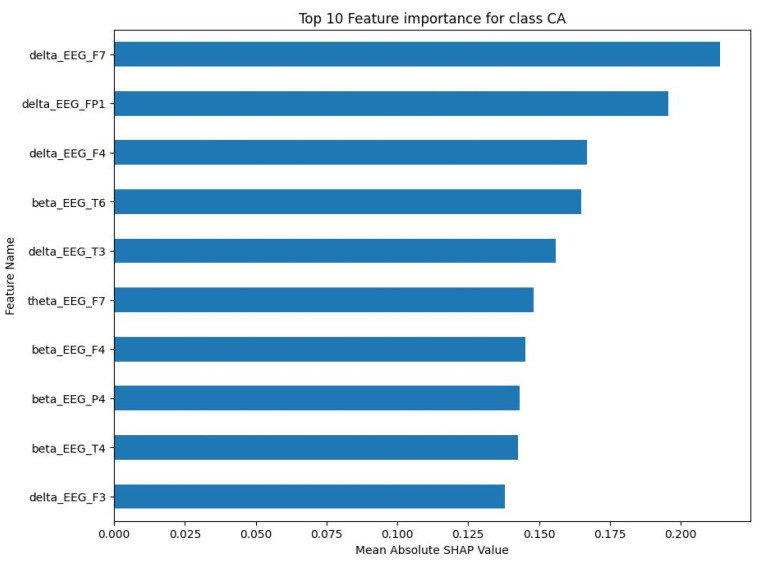
Top 10 important features for CA class.

**Figure 9:**
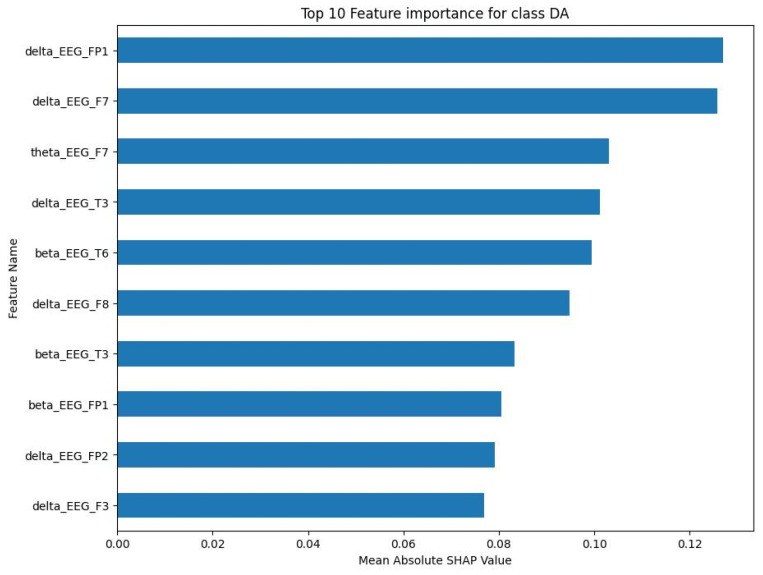
Top 10 important features for DA class.

For the NE class, the top 10 features were primarily delta and beta frequency bands from various EEG channels. The mean absolute SHAP values for these characteristics, as shown in Figure 6, ranged between 0.15 and less than 0.25. The SS class showed a similar pattern, with the top 10 features being predominantly delta and beta frequency bands. As shown in Figure 7, the mean absolute SHAP values for these characteristics ranged between a little less than 0.08 and a little bit less than 0.11. The CA class also showed a predominance of delta and beta frequency bands in the top 10 features. Figure 8 illustrates that the mean absolute SHAP values for these features ranged between 0.127 and approximately 0.225. Lastly, for the DA class, the top 10 features were primarily delta and beta frequency bands. Figure 9 shows that the mean absolute SHAP values for these features ranged between a little bit less than 0.08 and a little bit more than 0.12.

## 6. Discussion

The results of this study demonstrate the potential of using the proposed approach to detect mental states based on PSD features of the EEG data. The high-performance metrics across all pilots suggest that the model is effective in distinguishing between the four mental states: CA, DA, SS, and NE.

The use of PSD features from five frequency bands (i.e., delta, theta, alpha, beta, and gamma) across 20 EEG channels likely contributed to the model’s high performance. These features provide a rich representation of the EEG signals, capturing important frequency-specific information that is relevant for distinguishing between different mental states. PSD features encapsulate the power distribution over various frequency bands, which is a crucial aspect of EEG signals that are often linked to different mental states. However, the variation in the model’s performance across different pilots indicates that individual differences may have influenced the results. Each pilot may have unique EEG patterns and responses to different mental states, which could affect the model’s performance. For example, the model achieved the highest performance metrics with Pilot 2, suggesting that the features of this pilot’s EEG data were particularly well-suited to the model. On the other hand, the model’s performance was lowest with Pilot 12, indicating that there may be unique aspects of this pilot’s EEG data that were not as effectively captured by the model.

The fact that the model performed well on the combined data of all pilots is promising. It suggests that the model is capable of generalising across different individuals, which is crucial for its potential application in real-world settings. However, the slightly lower recall score in comparison to the other metrics indicates that there is still room for improvement in the model’s ability to correctly identify all instances of the different mental states. Future work could explore ways to further improve the model’s performance. This could include refining the model’s architecture, experimenting with different methods of preprocessing the EEG data or incorporating additional features that capture more information about the pilots’ mental states. For instance, exploring different types of feature extraction methods or incorporating temporal information could potentially enhance the model’s performance. Additionally, additional validation with larger datasets and in real-world settings would be beneficial to confirm these findings and further refine the model.

This study provides valuable insights into the potential of using the proposed framework for detecting mental states based on PSD features of EEG data. The high-performance metrics achieved by the model suggest that it could be a valuable tool in fields such as aviation, where monitoring pilots’ mental states could contribute to safety and performance. However, it is important to note that while the model’s performance is promising, the interpretation and application of these results should be carried out with caution. The model’s performance is based on the specific dataset used in this study, and its performance may vary with different datasets. Therefore, further research and validation are necessary to fully understand the model’s capabilities and limitations.

Analysis of the accuracy and loss curves provides valuable insights into the learning process and its ability to generalise from the training data to unseen data. The steady increase in the training accuracy and the consistent decrease in the training loss demonstrate that the model was effectively learning from the PSD features extracted from the EEG data. This suggests that the model’s architecture and the preprocessing steps taken, including the use of PSD features, were well suited for the task of detecting the four mental states.

The slight irregularities observed in the validation accuracy and loss curves suggest that the model’s performance varied when applied to different subsets of data. These fluctuations could be due to a variety of factors, including inherent variability in the EEG data, individual differences between pilots, or the specific division of the data into training and validation sets. Despite these irregularities, the overall positive trend in the accuracy of the validation and the general decrease in the validation loss indicates that the model was able to generalise its learning to new data, which is a crucial aspect of its performance. However, the presence of these irregularities also suggests potential areas for improvement in the model. Future work could explore different strategies for managing these irregularities, such as adjusting the model’s architecture, experimenting with different methods of data preprocessing, or using different strategies for dividing the data into training and validation sets. In conclusion, the accuracy and loss curves provide additional evidence of the potential of the proposed model to detect mental states based on PSD features extracted from the EEG data. Despite some irregularities in the validation curves, the overall trends suggest that the model is capable of learning effectively from the data and generalising its learning to new data. This holds promise for the model’s application in real-world settings, such as aviation, where accurate detection of pilots’ mental states could contribute to safety and performance.

The confusion matrix provides a deeper understanding of the model’s performance across the four mental states. It is evident that the model performs exceptionally well in classifying the SS and DA states, with almost perfect accuracy rates of 99.90% and 99.91%, respectively. This high level of accuracy suggests that the model is highly effective in distinguishing these states, likely due to the distinct PSD features associated with these mental states in the EEG data. The CA state also saw a high accuracy rate of 95.51%, indicating that the model is also capable of effectively identifying this state. However, the NE state had a noticeably lower accuracy rate of 81.94%. This could be due to the inherent complexity in distinguishing the NE state from the other mental states, as the NE state might not exhibit as distinct PSD features as the other states. The off-diagonal elements of the confusion matrix, which represent the instances where the model made incorrect predictions, provide further insights into the model’s performance. For instance, the model misclassified the NE state as the CA state in 12.67% of instances. This could suggest that the EEG features of these two states might share some similarities, causing the model to confuse between them. 

Despite these challenges, the overall performance of the model, as demonstrated by the confusion matrix, is highly promising. The model’s ability to achieve high accuracy rates across the four mental states suggests that it is capable of effectively using PSD features extracted from EEG data to detect different mental states. Future work could focus on improving the model’s ability to distinguish the normal state, potentially by incorporating additional features or refining the model’s architecture. Furthermore, additional validation with larger datasets and in real-world settings would be beneficial to confirm these findings and to further refine the model.

The results stated in Table 3 reveal a compelling narrative regarding the superior performance of the proposed framework in comparison to other well-established neural network architectures. The comparative analysis unveiled a marked improvement in classification accuracy by the framework model, thereby underlining its robustness and efficacy in deciphering intricate patterns within the data. Specifically, the substantial lead of 21% over the DCAEN model, 12% over the CNN model, and 16% over the LSTM and ANN models underscores the adeptness of the proposed framework in handling the inherent complexities of the classification task. The pronounced superiority in performance could be attributed to the careful design of the proposed framework, which possibly enabled a better understanding and representation of the underlying data distributions. Furthermore, the results accentuate the potential of the proposed framework for broader applicability in related domains demanding high-accuracy classification tasks. It is salient to acknowledge that while the proposed framework outperformed the other models, the comparative analysis also sheds light on the areas where other models could be refined and optimised for better performance.

Upon the analytical examination of the model performance, a salient feature of our model’s architecture merits discussion. Unlike frameworks employing recurrent layers such as LSTM layers, our model, anchored by 1D-CNN, availed a clear pathway to discern the contribution of individual features towards specific predictions. This clarity was further enhanced via the employment of the SHAP method, which enabled a detailed exploration into feature importance. The comparison of our model against those incorporating LSTM layers underscores a pervasive challenge: the inherent intricacy of recurrent mechanisms often veils the precise attribution of features, making the elucidation of individual feature contributions towards predictions a challenging endeavour. However, the integration of 1D-CNN and SHAP in our model mitigated this challenge, fostering not only superior classification accuracy but also augmented interpretability. This aspect of interpretability is crucial, especially in domains where deciphering the rationale behind model predictions is imperative for garnering trust and facilitating actionable insights.

The results of the SHAP analysis, as visualised in Figure 6, Figure 7, Figure 8 and Figure 9, provide valuable insight into the most important features to predict each mental state. The predominance of delta and beta frequency bands in the top 10 features for each class suggests that these frequency bands may be particularly important for distinguishing between different mental states. Delta waves are typically associated with sleep or deep relaxation, while beta waves are associated with active thinking or focus. This aligns with the nature of the mental states being predicted, as channelised and diverted attention would likely involve more active thinking (beta waves), while a normal state might be more relaxed (delta waves).

However, it is interesting to note that the specific EEG channels that were most important varied between the classes. This suggests that different mental states may be associated with activity in different regions of the brain, which is captured by the different EEG channels. For example, the F7 channel (frontal lobe) was important for the NE, SS, and CA classes, while the T3 channel (temporal lobe) was important for the SS and DA classes. This could potentially provide insights into the neural mechanisms underlying these mental states.

The range of the mean absolute SHAP values for the top 10 features in each class also provides information about the relative importance of these features. The NE and CA classes had higher SHAP values compared to the SS and DA classes, suggesting that the top features for NE and CA may have a stronger influence on the model’s predictions.

These findings highlight the complexity of predicting mental states based on EEG data and the importance of considering both the frequency and spatial information contained in the data. However, further research is needed to fully understand the implications of these results and to explore how this information can be used to improve the model’s performance. For instance, it might be beneficial to incorporate these findings into the feature selection or preprocessing stages of the model development process.

## 7. Conclusions

The present study has made significant strides in demonstrating the potential of the proposed approach for the classification of distinct mental states, specifically CA, DA, SS and NE states, based on PSD features derived from EEG data. The model’s performance, as assessed by accuracy, precision, recall, and F1 score metrics, was consistently high across all pilots, indicating its robustness and generalisability. This is a promising finding, suggesting that the model can effectively learn from individual pilots and apply this learning to a broader population.

The use of SHAP values in this study has provided a deeper understanding of the model’s decision-making process. By identifying the most influential features for each mental state, we have gained insights into the importance of both frequency-specific and spatial information in EEG data for mental state classification. The predominance of delta and beta frequency bands in the top features for each class suggests that these frequency bands play a crucial role in differentiating between various mental states.

However, the study also revealed the complexity of the task at hand. The variation in the model’s performance across different pilots and the range of SHAP values across classes underscore the influence of individual differences and the complexity of the mental states being predicted. These findings suggest that while the model is effective, there is still room for improvement and refinement. Future work could focus on enhancing the model’s architecture, exploring different preprocessing methods, or incorporating additional features that capture more nuanced aspects of the pilots’ mental states.

Furthermore, the findings need to be validated with larger datasets and in real-world settings to confirm their applicability and further refine the model. The high-performance metrics achieved by the model suggest its potential utility in fields such as aviation, where monitoring pilots’ mental states could contribute to safety and performance. However, the interpretation and application of these results should be carried out with caution, considering the specific dataset used in this study and the potential variability in the model’s performance with different datasets.

In conclusion, this study contributes valuable insights into the potential of CNN models for mental state detection based on PSD features of EEG data. It underscores the importance of comprehensive feature representation, the influence of individual differences, and the need for further research and validation to fully realise the potential of this approach. The findings of this study pave the way for future research in this area, with the ultimate goal of enhancing sty and performance in high-stakes fields such as aviation.

## Figures and Tables

**Table 1:** Hyperparameters of the layers of the 1D-CNN model.

Layer	Filter	Kernel Size	Activation Function	Padding	Kernel Initializer
1	64	3	Relu	Same	GlorotNormal
2	128	3	Relu	Same	GlorotNormal
3	256	3	Relu	Same	GlorotNormal
4	128	3	Relu	Same	GlorotNormal
5	64	3	Relu	Same	GlorotNormal

**Table 2:** Classification results of individual and combined pilots.

Pilot ID	Accuracy	Precision	Recall	F1 Score
1	97	97	95	96
2	99	99	98	98
3	97	98	95	96
4	96	94	94	94
5	97	98	96	96
6	97	96	94	95
7	97	98	96	96
8	96	95	92	93
9	97	96	97	97
10	95	96	90	92
11	95	95	89	91
12	94	91	89	90
13	96	95	93	94
14	96	97	92	93
15	97	95	96	95
16	98	98	95	96
17	98	97	96	97
All	96	96	94	95

**Table 3:** Comparative performance analysis of the proposed framework with established neural network models.

Model	Authors	Performance Accuracy	Relative Performance Improvement
DCAEN	[27]	75%	21%
CNN	[30]	84%	12%
LSTM	[21]	80%	16%
ANN	[20]	80%	16%
Proposed Model	-	96%	-

## Data Availability

The input data, source code, and output data can be found via the DOI link: https://doi.org/10.17862/cranfield.rd.24155832 (accessed on 6 November 2023).
